# The hazards of genotype imputation when mapping disease susceptibility variants

**DOI:** 10.1186/s13059-023-03140-3

**Published:** 2024-01-03

**Authors:** Winston Lau, Aminah Ali, Hannah Maude, Toby Andrew, Dallas M. Swallow, Nikolas Maniatis

**Affiliations:** 1 Department of Genetics, Evolution and Environment, UCL Genetics Institute, University College London, London, UK; 2 Department of Metabolism, Digestion and Reproduction, Section of Genetics and Genomics, London, UK

## Abstract

**Background:**

The cost-free increase in statistical power of using imputation to infer missing genotypes is undoubtedly appealing, but is it hazard-free? This case study of three type-2 diabetes (T2D) loci demonstrates that it is not; it sheds light on why this is so and raises concerns as to the shortcomings of imputation at disease loci, where haplotypes differ between cases and reference panel.

**Results:**

T2D-associated variants were previously identified using targeted sequencing. We removed these significantly associated SNPs and used neighbouring SNPs to infer them by imputation. We compared imputed with observed genotypes, examined the altered pattern of T2D-SNP association, and investigated the cause of imputation errors by studying haplotype structure. Most T2D variants were incorrectly imputed with a low density of scaffold SNPs, but the majority failed to impute even at high density, despite obtaining high certainty scores. Missing and discordant imputation errors, which were observed disproportionately for the risk alleles, produced monomorphic genotype calls or false-negative associations. We show that haplotypes carrying risk alleles are considerably more common in the T2D cases than the reference panel, for all loci.

**Conclusions:**

Imputation is not a panacea for fine mapping, nor for meta-analysing multiple GWAS based on different arrays and different populations. A total of 80% of the SNPs we have tested are not included in array platforms, explaining why these and other such associated variants may previously have been missed. Regardless of the choice of software and reference haplotypes, imputation drives genotype inference towards the reference panel, introducing errors at disease loci.

**Supplementary Information:**

The online version contains supplementary material available at 10.1186/s13059-023-03140-3.

## Background

Genotype imputation is a statistical technique that infers missing data by assigning the most likely genotypes for SNPs that have not been directly genotyped using a collection of allelic combinations (haplotypes) from a very large number of individuals, known as the haplotype reference panel. This haplotype reference panel, typically from whole genome sequence, is then used to impute into the study panel at a subset of non-genotyped SNPs by finding haplotype segments that are shared between the study and reference panels. This popular technique has become a standard tool [1] in candidate gene studies, in genome-wide association studies (GWAS), and their meta-analysis, to maximise the number of SNPs which can be tested for association by overcoming the varied SNP coverage across studies. The wide acceptance of using imputed (in silico) SNPs is thus justified by the need to increase power through the substantial increase in marker density (typically using hundreds of thousands of genotyped SNPs in the study to impute millions of missing study SNPs in the final analysis) and the ability to meta-analyse identical SNPs across different array platforms. But interrogating the contexts in which the accuracy of imputation is compromised requires closer investigation, since most studies that have examined imputation performance have considered genome-wide error rate and focussed on ways to improve imputation errors by comparing different software, data, and reference panels. But detailed investigations on the performance of imputation at specific gene regions or disease-associated loci in relation to haplotype structure are lacking.

We recently examined the impact of imputation in a chromosomal region under positive selection [2]. Variants within the region of the well-known lactase gene (*LCT*), which have become frequent in a limited number of modern populations due to selection, were studied by comparing imputed and directly observed genotypes. The majority of incorrectly assigned and failed genotype imputations in this context were observed to be a result of long haplotypes that are evolutionarily closely related to those carrying the derived alleles [2] but were also due to rare and recombinant haplotypes. We concluded not only that signatures of selection can decrease the accuracy of imputation but also that in most cases there was an allelic bias for the imputed and missing assignments.

Here, we investigate the impact of imputation on mapping disease susceptibility variants. The accuracy of imputation in association mapping of disease loci is of particular importance when interpreting GWAS results but also polygenic risk scores as they too are based on imputed SNPs. In addition, it is now well-known that association signals identified in European-GWAS show low reproducibility in other ethnic populations despite similar disease prevalence. Currently, imputation is used across the board, from the integration of multiple types of data for colocating signals, fine mapping of candidate genes, to plant and animal breeding studies [3] where genomic analyses for economically important traits are further complicated by the impact of long-term artificial selection.

Therefore, and following up from our previous work on imputation in relation to selection, studying the accuracy of imputation in the context of gene mapping is as topical as ever, and questioning the role of imputation from different and new perspectives is paramount. This study is motivated by the desire to (a) use a more realistic study design to investigate imputation by assigning multiple statistically significant markers ‘missing’ as opposed to masking one marker at a time, (b) to reconstruct haplotypes for the case–control study panel and compare them with the haplotypes from the reference panel in order to identify how and why imputation errors arise, and (c) to use candidate disease loci as opposed to genome-wide data because differences in haplotype frequencies are expected to arise at disease loci and also because more accurate haplotypes can be determined for smaller regions  [4].

The three loci we are examining for imputation accuracy herein were chosen from our previously reported transethnic study [5] on type-2 diabetes (T2D) in Europeans and African-Americans using a multi-SNP association method that obviated the need for imputation. Instead of reporting lead SNPs, this methodology directly estimates the locations of disease susceptibility variants on genetic maps with distances expressed in linkage disequilibrium units (LDU maps). Hence, the previous study [5] was powered by incorporating population-specific patterns of linkage disequilibrium (LD) in the association mapping analyses. Two of the three loci, *ACTL7B* and *KCNK3*, were selected for investigating imputation because they were first reported by us [5] but had not been identified in other GWAS, and in addressing why this might be the case, we speculated that associations might be missed through inaccuracy of imputation. The third locus, *TCF7L2*, was selected because it is a well-established and most potent T2D signal that we (without imputation) [5] and many others (with imputation) had identified. Hence, the latter is used as benchmark in the present imputation study. For all three loci, we are using previously available fine mapping data (targeted next-generation sequencing, NGS).

## Results

### Study data, reference panel and haplotypes

The performance of imputation was investigated using NGS data in T2D cases and controls for the three T2D susceptibility loci, *ACTL7B*, *KCNK3*, and *TCF7L2*, with lengths of 40, 41, and 8 kb, respectively (Table 1). Andres et al. [4] have shown that phasing performance is the most accurate with segments shorter than 50 kb making these three regions good candidates for investigating the impact of haplotype structure on the quality of imputation. Also, the SNP density used for this study is 3–5 times higher than standard SNP arrays that have been used in meta-analyses and even the more recent 2.5-M SNP array (Table 1). The available NGS data of European ancestry have been previously reported elsewhere [5], and in the “Methods” section herein, but in brief, these included 92 T2D cases with a family history of T2D and 93 selected controls without a history of T2D.

**Table 1:** Characteristics of the three gene regions studied. Total number of SNPs in the study panel includes the masked SNPs (23 T2D associated) and scaffold SNPs. The density of the study’s experiments is compared with the density of two second-generation genotyping platforms with approximately 1 and 2.5 million SNPs

Gene regions	*ACTL7B*	*KCNK3*	*TCF7L2*
Chromosome	9q	2p	10q
Genomic location in bp (B37)	111550543–111589986	26894433–26935356	114740628–114748940
Distance in kb	39.4	40.9	8.3
Total SNPs in the NGS study (197)	118	59	20
Total significant T2D-associated SNPs (23)	7	8	8
***Density (average number of SNPs per 1 kb)***			
Our NGS study panel	3.0	1.5	2.4
Affymetrix 6.0 1-M SNP array	0.7	0.3	0.5
Illumina Omni 2.5-M SNP array	0.9	0.7	0.7

**Fig. 1:**
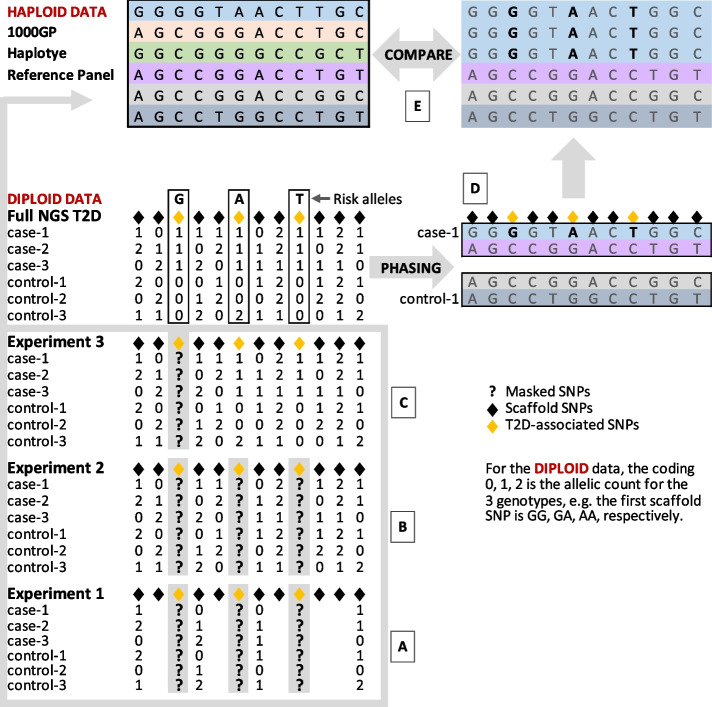
Study design schema for the imputation experiments (bottom to top A–E). T2D-associated SNPs were masked simultaneously and imputed using scaffold marker SNPs at **A** low density and **B** high density based on all the remaining NGS data, while **C** masked one T2D-associated SNP at a time. **D** The full NGS data was phased independently using PHASE v2.1.1, and **E** the haplotype frequencies in cases and controls were compared with those from the 1000GP haplotype reference panel

The total number of significant SNPs (*P* < 0.05) from all three gene regions with evidence of association with T2D is 23. These associations are based on allelic *χ*^2^ tests for each SNP in the real targeted NGS data (Table 1). The study investigated the performance of genotype imputation by ‘masking’ these 23 significant SNPs (i.e. removing them from the data) and imputing them using the remaining NGS SNPs as ‘scaffolds’ via three experiments, as shown in the schematic Fig. 1A–C. Experiments 1 and 2 masked all 23 T2D-associated SNPs simultaneously and imputed them using the scaffold SNPs at low and high coverage, respectively. The low-coverage experiment represents the low SNP density of the early generation GWAS arrays, such as the 500-k Affymetrix used for T2D by the WTCCC [6]. The high coverage in experiment 2 used all the remaining SNPs in the NGS study for these 3 loci ( 197–23 = 174) with average density much higher than the more recent second-generation genotyping arrays (Table 1). The final experiment 3 used the high-density 174 scaffold SNPs but masked the 23 T2D-associated SNPs one at a time rather than simultaneously for comparison with previous studies of imputation quality. Note that the *TCF7L2* region was excluded from the first sparse marker panel experiment due to its shorter kb length than the *ACTL7B* and *KCNK3*.

Imputation was carried out using IMPUTE2 [7], and the phase 3 global haplotypes from the 1000 Genomes Project (1000GP) [7] were used as our main reference panel to impute genotypes for the masked SNPs. The 1000 GP has been used by most GWAS to date, including the two latest large-scale T2D studies [8, 9]. For the low-coverage experiment 1, we also used the case–control T2D NGS as the reference panel. Single SNP association analyses (allelic *χ*^2^ tests) were carried out for both the observed and inferred genotypes of all the T2D-associated SNPs using PLINK [10].

**Fig. 2:**
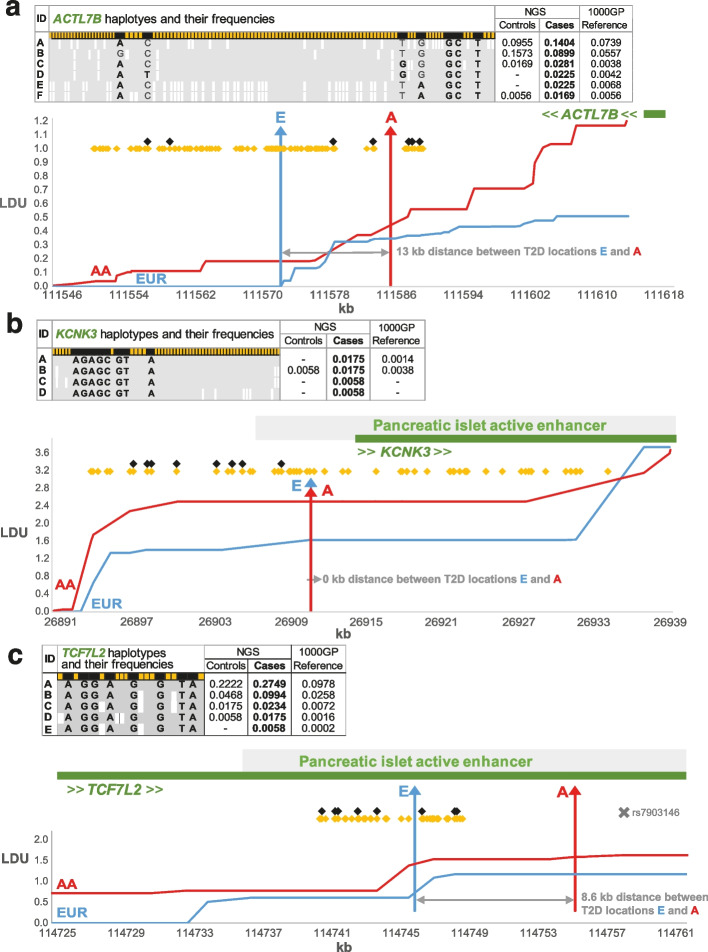
Locations of the imputed SNPs in relation to the T2D locations and surrounding haplotypes. The tables for the a) *ACTL7B*, b) *KCNK3* and c) *TCF7L2* regions give the frequencies of the haplotypes using all ♦scaffold and ♦T2D-associated (imputed) SNPs with their risk alleles in bold. The white bars within the tables represent other allelic differences between the haplotypes. The T2D locations, **A** and **E** were estimated [5] from African-American (AA) and European (EUR) data and mapped onto the respective LDU maps (linkage disequilibrium distances on Y axis). The published GWAS lead SNP (rs7903146) for *TCF7L2* is also plotted in Fig. 1c

In addition, the NGS 92 cases and 93 controls were independently phased as one group (Fig. 1D) using PHASE v2.1.1 [11] in order to conduct detailed comparisons of haplotypes between the study and reference panels (Fig. 1E). Results of the analyses on haplotype structure are thus independent from those of the imputation analyses and allowed us to identify the reasons for errors that arose. The LD structure for the three gene regions studied is also shown by plotting the genetic locations of all SNPs in linkage disequilibrium units (LDU) on their physical locations in kb. Two high-resolution LDU maps were constructed based on the 1000GP European (EUR) and African-American (AA) SNP datasets (Fig. 2).

### Comparisons between observed and imputed genotypes

Table 2 presents the summary statistics for all the significant SNPs for the three comparison experiments. The first experiment, based on the low-resolution study panel with all significant SNPs masked, produced the worst outcome as none of these SNPs was accurately imputed. All but two of the SNPs across loci were imputed as monomorphic. For markers with low MAF of 0.01 (e.g. rs144726287, *ACTL7B*), incorrect genotype calls were sufficient to impute them as monomorphic, but other SNPs with higher MAF were inferred as monomorphic due to missingness as well as incorrect genotyping, mainly for the risk allele (e.g. *ACTL7B* rs72756001 with *MAF* = 0.08, Additional file 1: Table S1). This was despite the high reported info score for this marker (0.72). The only two SNPs that remained polymorphic after imputation (*ACTL7B* SNPs rs10979533, rs12380226) were in perfect LD with each other, and despite their exceptionally high info score of 0.95, which implies near perfect inference, both were imputed incorrectly and with type 2 errors. The result was that these two *ACTL7B* SNPs were no longer significantly associated with phenotype (*χ*^2^ of 0.009 instead of the expected *χ*^2^ of 4.131, Table 2), primarily due to very large number of incorrectly imputed genotypes but also few missing genotypes for the risk allele (Additional file 1: Table S1).

**Table 2:** Results from imputation under different experiments. The T2D-association statistic (*χ*^2^) for the observed and imputed SNPs using low- and high-marker densities. SNPs with ‘-’ were imputed as monomorphic. The first experiment was not feasible for *TCF7L2*. Significant SNPs were masked simultaneously (second experiment) or one at a time (third experiment)

3 T2D loci studied with the NGS data on 92 T2D cases and 93 controls							First experiment		Second experiment		Third experiment	
							**Low density**		**High density**		**High density**	
SNPs	Major/minor	Risk	MAF NGS EUR	MAF 1000GP		Observed NGS *χ*^2^	Imputed *χ*^2^	Info score	Imputed *χ*^2^	Info score	Imputed *χ*^2^	Info score
				EUR	Global							
***ACTL7B***												
rs13285616	A/G	A	0.13	0.14	0.06	5.11	-	0.05	-	0.18	-	0.17
rs144726287	C/T	T	0.01	0.01	0.00	4.09	-	0.00	-	0.07	-	0.07
rs60388922	T/G	G	0.10	0.08	0.07	4.37	-	0.06	5.69	0.95	5.08	0.97
rs72756001	G/A	A	0.08	0.09	0.12	4.66	-	0.72	4.97	0.98	4.97	0.98
rs10979533	G/T	G	0.08	0.07	0.09	4.13	0.01	0.95	4.13	1.00	4.13	1.00
rs117056494	C/T	C	0.01	0.01	0.00	4.00	-	0.03	-	0.06	-	0.06
rs12380226	T/C	T	0.08	0.07	0.09	4.13	0.01	0.95	4.13	1.00	4.26	0.99
***KCNK3***												
rs78489206	G/A	A	0.03	0.05	0.05	4.67	-	0.06	3.91	0.82	3.91	0.97
rs7588614	C/G	G	0.04	0.05	0.12	3.99	-	0.13	5.60	0.81	5.60	1.00
rs77786658	G/A	A	0.03	0.05	0.05	4.84	-	0.06	4.67	0.82	4.67	1.00
rs6707973	A/G	G	0.04	0.05	0.17	3.99	-	0.27	3.99	0.85	3.99	0.99
rs73920335	T/C	C	0.04	0.05	0.17	3.99	-	0.29	3.83	0.91	3.83	0.97
rs59435210	T/G	G	0.04	0.05	0.17	3.99	-	0.30	3.24	0.89	3.24	0.98
rs59284336	C/T	T	0.03	0.05	0.04	4.67	-	0.09	4.75	1.00	4.75	0.98
rs113076024	G/A	A	0.03	0.05	0.04	4.75	-	0.09	4.67	1.00	4.67	1.00
***TCF7L2***												
rs7081062	A/G	A	0.32	0.36	0.34	9.96			-	0.56	9.32	1.00
rs149954646	G/A	G	0.01	0.03	0.01	4.00			4.00	0.61	4.00	1.00
rs145034729	G/A	G	0.01	0.03	0.01	4.00			4.00	0.61	4.00	1.00
rs10128255	A/G	A	0.32	0.36	0.34	9.32			-	0.56	9.14	1.00
rs7895307	G/A	G	0.32	0.36	0.34	9.32			-	0.56	9.32	0.99
rs4073980	G/C	G	0.46	0.50	0.44	6.85			0.04	0.75	7.50	0.99
rs4074720	T/C	T	0.46	0.50	0.44	6.85			0.41	0.76	6.85	1.00
rs4074718	A/G	A	0.46	0.50	0.44	6.85			0.41	0.77	6.85	1.00

The second experiment using the high-resolution study panel increased the number of imputed genotypes (Table 2). Nevertheless, the quality of imputation was still poor overall with three noteworthy results:

1. Despite the very large increase of scaffold SNPs, some SNPs were still imputed as monomorphic and only few (5/23) imputed genotypes across the 3 loci matched the observed ones;
2. Despite the high info scores, > 0.75 for the last 3 markers of *TCF7L2* and > 0.85 for the first 3 of the *KCNK3*, markers were imputed at high discordance with the observed genotypes due to a combination of missing and incorrect calls, mainly for the risk allele at both loci. These SNPs, within each of the two genes, are in perfect LD in the 1000GP European population (CEU), but nevertheless, their LDU genetic distance is not zero (i.e. there is some LD breakdown, see Fig. 2B, 2C). This difference is detected because the LDU map considers all informative pairwise associations in a gene region to calculate genetic distances, not just pairwise analyses of the three SNPs.
3. Finally, the *KCNK3* rs59435210 is flanked on either side by well-imputed SNPs (rs73920335 and rs59284336), but incorrect imputation still yielded a false-negative association with this SNP. These SNPs are in an LDU block for the African ancestry population, but rs59435210 is on a different block on the European LDU map (Fig. 2B). The MAFs for these SNPs were very similar between the European NGS and the European 1000GP populations (0.04 and 0.05, respectively) but different from the reference global panel for which these markers are much more common (MAF of 0.17).

Masking all the significant SNPs at once for this experiment represents a realistic scenario as the vast majority (80%) of these were found to be missing in the second-generation large-scale genotyping arrays (e.g. Illumina Omni 2.5 M, Affymetrix 6.0 1 M). In particular for *ACTL7B*, none of the SNPs we tested are included on these GWAS platforms or on the Metabochip, which is a custom SNP array designed to follow up large-scale genotyping studies.

The third experiment involved the masking of significant SNPs one at a time rather than simultaneously. As expected, this analysis produced the best quality of imputed genotypes. With the exception of three *ACTL7B* SNPs that remained monomorphic, all other markers were imputed with almost perfect info scores (> 0.96, Table 2) and high concordance between the in silico and observed genotypes, giving an overall accuracy of ~ 90%. Of the three monomorphic SNPs, the risk allele for rs13285616 (*MAF* = 0.13) was missing from both cases and controls. But for the two rarer ones with *MAF* = 0.01, the risk allele of rs144726287 was incorrectly assigned the alternative allele in the cases, and the alternative allele of rs117056494 was incorrectly assigned the risk allele in the controls (Additional file 1: Table S1). Overall, we did not notice any obvious relationship between MAF and quality of imputation. For example, imputation of *TCF7L2* SNPs with the same MAF of 0.01 yielded identical results to the observed genotypes.

However, the high quality of imputation for this third analysis does not represent a real-world GWAS nor does it provide insight into the process of imputation. The striking differences between masking one SNP at a time versus masking several simultaneously required closer examination. To help elucidate this, we examined and compared the haplotypes from the study panel in relation to the haplotypes from the reference panel (1000 GP). To our knowledge, comparisons of haplotype frequencies between the study- and reference- panels have not been previously investigated, but we consider this an essential analytical strategy because regardless of the imputation method used (e.g. IMPUTE2, minimac4, Beagle4.1), the same principle applies to all, whereby many variants that are not directly genotyped are being assigned inferred alleles based on a global collection of haplotypes from a high-resolution reference panel. The high-resolution NGS study panel from the second experiment was used because we wanted to examine haplotype frequencies under the best scenario possible in terms of marker density.

### Comparisons of haplotypes between the cases and the reference panel

For each of the three T2D loci, haplotypes were independently reconstructed for the pooled NGS study data using PHASE. Haplotypes carrying risk alleles of the T2D-associated SNPs were selected for further analysis with the aim of comparing their frequencies between the cases and controls and with the frequencies of the identical haplotypes from the 1000GP reference panel (Fig. 2). For *KCNK3* and *TCF7L2*, all the risk alleles co-segregated and tagged a set of highly similar haplotypes indicating a monophyletic origin (single founder). For *KCNK3*, all four risk haplotypes were found to be more common in cases compared to both the controls and global reference data. Two haplotypes in particular (A and B) were much rarer in the global reference panel (Fig. 2B). *TCF7L2* gave very similar results, with all five risk haplotypes being much rarer in the global and NGS control data than in cases (Fig. 2C). Haplotype A in particular showed a more than double the frequency in the NGS cases (0.27) and in the NGS cases and controls combined (0.24) compared to the global reference panel (0.098).

Unlike the other two genes, *ACTL7B* yielded six risk haplotypes with varying combination of risk alleles at different haplotypes, demonstrating the more complex and polyphyletic origin. Recurrent mutation [12], admixture, recombination, and gene conversion can produce such polyphyletic haplotypes [13], and here, they seem to complicate imputation further. In the case of *ACT7B*, there has clearly been recombination as shown by the breakdown of LD on the genetic map (Fig. 2A). *ACTL7B* is the only locus with failed imputed SNPs (Table 2) even for the third experiment (masked one at a time). Like the other loci though, the same finding was replicated with the *ACTL7B* where haplotypes were found to be rarer in the reference panel than in the T2D cases.

## Discussion

Imputation is used as a tool for increasing the power of GWAS as well as facilitating fine mapping of candidate loci [14]. It also enables increased coverage of low-depth whole-genome sequencing allowing this to be used as an alternative to SNP arrays [15, 16]. However, our study demonstrates that imputation can be inaccurate when searching for disease risk alleles which differ in frequency in cases compared with controls and reference panels, resulting in an imputation bias against the risk allele and consequently false-negative results. Indeed, other studies have also shown this bias, which results in a decrease of the association effect size [17], and have speculated that differences in the haplotype structure between study and reference panels might provide an explanation [17].

We thus have some sobering messages for those seeking to impute. The results presented in Fig. 2 show experimentally for the first time that when haplotypes carrying the risk alleles are considerably less common in the reference panel than in the cases included in the study, imputation distorts genotype inference towards the global reference panel. This novel finding was replicated in all the three gene regions we studied. Imputation is typically based on haplotypes constructed from a random cohort (e.g. 1000GP). The high-coverage whole-genome sequencing from the Trans-Omics for Precision Medicine (TOPMed) [18] programme provides a much larger reference panel than the 1000GP. Interestingly, recent studies [19] have shown improved quality of TOPMed imputation for rare SNPs (< 0.01) across the genome. But still, TOPMed imputation quality was found to vary widely, with regions not well imputed across ancestries even at > 0.05 MAF [19]. This finding supports the need for our study which was designed using short but disease-associated regions, to elucidate why many of the key SNPs are inaccurately imputed and hence give rise to false-negative tests of association. Even larger and more diverse reference panels will not overcome the problem of differences in prevalence between an ascertained case study and the overall haplotype frequencies of a reference panel. Hybrid panels that combine custom reference and public reference panels [1] or small samples of ethnically matched disease-specific cases and controls as reference panels [17] have been suggested as ways of improving accuracy of imputation. But when we used our own high-density NGS case–control data on T2D as the reference panel instead of 1000GP and with the low SNP-density scaffold SNPs as the study, we did not observe any major improvement in genotype errors compared to 1000GP (Additional file 1: Table S2). This is because, unsurprisingly, differences in haplotype frequencies still exist between the pooled T2D data and the frequencies in the cases carrying risk alleles at disease susceptibility loci. In fact, it is these differences that give rise to true-positive associations. Furthermore, the strategy of obtaining disease-specific reference panels is questionable, not only because of our findings but also because it would defeat the purpose of imputation if disease- and population-specific reference panels are required for every polygenic disease and trans-ancestry GWAS.

The demonstration of improved imputation by increasing study panel density is consistent with previous findings [7, 20, 21]. Here, we additionally demonstrate that SNPs with high certainty info scores (> 0.7 and > 0.9) in the *ACTL7B* region are incorrectly imputed even though our ‘low SNP-density analysis’ had marker coverage far higher than the suggested > 200/Mb [21] for optimum performance. This is of concern since current published meta-analyses include multiple first-generation GWAS arrays with low density and filtering criteria including info scores of > 0.5 for T2D [9] or even using thresholds as low as > 0.3 in other studies [22]. Even after provision of much higher density in the study panel, genotypes may not be adequately imputed for certain SNPs across the MAF spectrum (common, intermediate, and rare) despite info scores of > 0.7.

Focusing on three disease loci instead of genome-wide error rates has allowed us to examine imputation in the context of haplotypes and to compare the findings from large-scale meta-analyses of T2D (Table 3). *ACTL7B* in particular is an interesting case because the significant SNPs presented herein are not included in any SNP genotyping arrays, including the second-generation platforms such as Illumina Omni 2.5 M and Affymetrix 6.0, which makes it comparable with our masking experiments. The summary statistics published in 2020 by the Million Veteran Program [8] based on transethnic T2D GWAS of 1.4 million participants show only one SNP (*ACTL7B*, rs117056494) at nominal significance level with no information on the remaining SNPs within *ACTL7B* and *KCNK3* (Table 3). The latest transethnic study on T2D in 2022 [9] meta-analysed more than 120 GWAS of ~ 181,000 cases and 1,160,000 controls from multiple populations. Again, the seven *ACTL7B* SNPs were not directly observed but imputed as nonsignificant (Table 3).

**Table 3:** Comparison of results with the published T2D large-scale meta-analyses. *P*-values and odds ratios from two independent studies [8, 9] that used imputation. Odds ratios (*NA*) in the NGS study [5] cannot be estimated because the allele is only present in either cases or controls. For the 2020 study, (ns) were nonsignificant but with no *P*-values provided. For the 2022 study, (ns) were imputed but nonsignificant with the exception of one (?) SNP that could not be imputed

			**Directly observed from the NGS study** [5]			**2020** **Meta-analysis** [8]		**2022** **Meta-analysis** [9]	
SNPs	Risk	Location (bp)	*χ*^2^	*p*-value	Odds ratio	*p*-value	Odds ratio	*p*-value	Odds ratio
***ACTL7B***									
rs13285616	A	111556995	5.11	2.4E-02	2.04	Ns		Ns	0.99
rs144726287	T	111559654	4.09	4.3E-02	*NA*	Ns		Ns	0.97
rs60388922	G	111579228	4.37	3.7E-02	2.09	Ns		?	?
rs72756001	A	111583976	4.66	3.1E-02	2.40	Ns		Ns	1.00
rs10979533	G	111588286	4.13	4.2E-02	2.22	Ns		Ns	1.01
rs117056494	C	111588683	4.00	4.6E-02	*NA*	1.1E-03	1.11	Ns	1.11
rs12380226	T	111589552	4.13	4.2E-02	2.22	Ns		Ns	1.00
***KCNK3***									
rs78489206	A	26897778	4.67	3.1E-02	4.73	Ns		Ns	1.00
rs7588614	G	26898869	3.99	4.6E-02	3.51	Ns		Ns	1.00
rs77786658	A	26899221	4.84	2.8E-02	4.85	Ns		Ns	1.00
rs6707973	G	26901215	3.99	4.6E-02	3.51	Ns		Ns	1.00
rs73920335	C	26904377	3.99	4.6E-02	3.51	Ns		Ns	1.00
rs59435210	G	26905580	3.99	4.6E-02	3.51	Ns		Ns	1.00
rs59284336	T	26906436	4.67	3.1E-02	4.73	Ns		Ns	1.00
rs113076024	A	26909473	4.75	2.9E-02	4.79	Ns		Ns	1.00
***TCF7L2***									
rs7081062	A	114740745	9.96	1.6E-03	2.04	2.8E-69	1.07	9.2E-66	1.07
rs149954646	G	114741507	4.00	4.6E-02	*NA*	1.9E-24	1.21	6.5E-12	1.19
rs145034729	G	114741673	4.00	4.6E-02	*NA*	2.0E-24	1.21	6.4E-12	1.19
rs10128255	A	114742835	9.32	2.3E-03	2.00	3.4E-68	1.07	4.3E-66	1.07
rs7895307	G	114743961	9.32	2.3E-03	2.00	6.8E-71	1.07	1.2E-67	1.07
rs4073980	G	114746580	6.85	8.9E-03	1.73	3.5E-185	1.18	2.6E-213	1.18
rs4074720	T	114748497	6.85	8.9E-03	1.73	4.4E-185	1.18	2.4E-214	1.18
rs4074718	A	114748617	6.85	8.9E-03	1.73	2.2E-185	1.18	7.8E-215	1.18

By contrast, using only two GWAS arrays of European and African ancestry, without imputation [5], we replicated a genome-wide significant causal location within the *ACTL7B* region for both populations that closely overlapped with an adipose eQTL for the distal *cis*-gene *EPB41L4B* [5]. The recent T2D Million Veteran Program (MVP) study did identify a GWAS signal within *EPB41L4B* [8] and subsequently replicated in the 2022 study but within a very long interval of 1 Mb [9]. We therefore should also consider the fact that our findings for *ACTL7B* and *KCNK3* were based upon much finer scale replication co-localisations (13 and 0 kb, respectively) between T2D studies of different ancestries based on their population-specific genetic LDU maps, which may also have contributed to their earlier discovery [5] without the use of imputation.

Similar results were obtained when imputing our significant interval in the *TCF7L2*, which is 10 kb away from the published lead SNP (rs7903146). This is a region of strong LD with limited haplotype diversity, and a few of the masked SNPs are included on GWAS/Metabochip arrays. Nevertheless, the results demonstrate the lack of sufficient power of the imputation methods to identify new variants within a well-studied and significant T2D locus and is consistent with other studies [20]. The 2014 meta-analysis identified only three of the eight significant *TCF7L2* SNPs we studied here, and only the 2022 meta-analyses (which uses ~ 1.5-M cases/controls) identify all eight as highly significant (Table 3). This supports our findings that the presence of T2D-associated scaffold SNPs in the datasets of the study panel does improve imputation. Indeed, masking one SNP at a time improved all results for all three regions, even for the *ACTL7B* locus with its more diverse set of background haplotypes. Therefore, the choice of the masking strategy in studies that examine imputation will have significant impact on the findings. It is likely that the ‘leave-one-out at a time’ approach or other masking approaches is one of the reasons behind the high-accuracy estimates of previous assessments on imputation. Another reason is that many of these used large datasets that are not specific to disease loci (i.e. no ascertained cases) with no anticipated haplotype differences between study and reference panels to impact upon the accuracy of imputation.

**Fig. 3:**
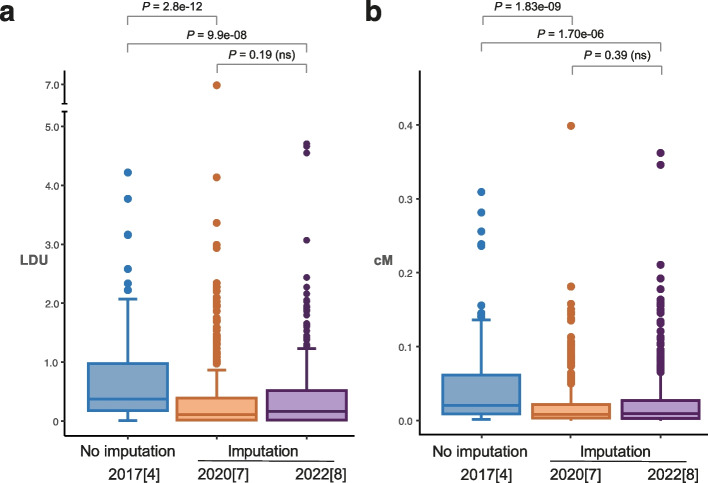
Comparison of genetic distances at T2D loci with or without imputation. Box plots of genetic distance across ± 10 kb from T2D-associated genetic loci, measured in **a** linkage disequilibrium units (LDU, European) and **b** centimorgan (cM) from the 1000 Genomes Project. Distances are plotted from loci mapped with no imputation (111 [5] loci) and loci mapped using imputation (459 [8] and 277 [9] loci). Lower median values (stronger LD) are observed in imputation studies. *P*-values from the Wilcoxon rank test statistic

To further investigate recent large-scale T2D GWAS that used imputation, we tested the extent of LD around all published lead SNPs identified to date by the large-scale transethnic meta-analyses in 2020 [8] and an independent one in 2022 [9]. We compared these with the LD surrounding the T2D signals identified without imputation [5] (Fig. 3). The GWAS studies that examine SNP associations and include imputation tend to identify disease loci in regions with more extended LD than loci identified with our multi-SNP LDU mapping approach that does not use imputation (Fig. 3). Regions of strong LD are more likely to contain some directly genotyped markers in the GWAS because array platform strategy is to tag LD blocks. But the implication of this observation is that missing genotypes are also more likely to be correctly imputed in regions of very strong LD. This is partly because haplotypes in the reference panel can be reconstructed with less error in such regions[4].

Another question is whether the odds ratios (ORs) from imputed SNPs in these LD regions are accurate. Comparing the ORs of the same studies in 2020 [8] and 2022 [9], we find that these are considerably lower than the ORs for the same directly observed SNPs that we genotyped in our NGS study. For example, the substantially increased number of T2D cases and controls that were meta-analysed for *TCF7L2* led to correspondingly low *P*-values but with ORs still close to 1.0 (Table 3), while our OR estimates for the same *TCF7L2* SNPs ranged from 1.73 to 2.04. This is also true for the previously reported, lead SNP rs7903146 within *TCF7L2*, which is included on some array platforms but not all. One of the first T2D GWAS [23] with no imputation, but also candidate-gene studies [24, 25] that directly genotyped the lead rs7903146, estimated much higher ORs (1.50–2.15) compared to the recent meta-analysis in Table 3 that was based on imputation [9]. It is therefore reasonable to conclude that even minor imputation errors can lead to considerably reduced effect size estimates.

Provided favourable assumptions hold, imputation is likely to perform well in population cohorts, but for disease-specific loci, problems of fine mapping and meta-analysing different GWAS will always remain, even in regions of strong LD. This is because of the variation in LD, and hence in haplotype structures, which is expected in the same regions but different populations. This could explain the common observation made by the community about the low reproducibility of lead SNPs across ancestries. Therefore, future work should investigate imputation in relation to LD, recombinational and genomic history, but also in other contexts, for example, in relation to other response variables such as RNA expression for mapping eQTLs. In addition, further consideration should be given to the accuracy of long-range phasing, which is another factor that impacts heavily upon imputation accuracy. The haplotypes in the reference panels (e.g. 1000GP or TOPMed) are inferred from unrelated individuals. Thus, the length of the genomic region used in phasing and the intensity of historical recombination (increased haplotype diversity), which varies across populations, will inevitably have a major impact upon the construction of haplotypes for any phasing and imputation software.

## Conclusion

Here, we show that haplotype differences between the study panel and the reference panel drives imputation towards making allelic calls that are more frequent in the reference panel, than in the case–control study. Therefore, searching of susceptibility variants using imputation is likely to produce inaccurate genotypes where it matters most, that is to say, in regions where haplotype differences between cases and controls are expected due to true-positive associations. This effect will be present regardless of how big and diverse the reference panel is; hence, we advocate methods which test for association without imputation. Here, we find that these errors lead to false-negative results, but equally, large differences in haplotype frequencies between cases and reference data could in some cases have other effects. The problems with imputation for mapping causal variants, together with previous findings on its inaccuracy in regions of positive selection [2], suggest the need for a shift in designing ways to integrate directly observed association signals, rather than imputing millions of SNPs with the hope they will perform equally well across different populations and omics data.

## Methods

### Study samples, genotyping, and T2D-associated and scaffold SNPs

Sequencing was conducted using the Agilent SureSelect^XT2^, on 92 T2D cases with a family history of T2D and 93 selected controls without a history of T2D. Cases and controls were from the same population of European ancestry and matched for age, BMI, and sex. At the time of publication [5], sequence reads were aligned to the human reference sequence (hg19/build37) using Burrows-Wheeler transform [26]. Post-alignment QC, variant calling, and QC were conducted according to Genome Analysis Toolkit guidelines [27]. SNP data were further filtered for having a minor allele frequency (MAF) above 1%, deviations from Hardy–Weinberg equilibrium in the controls only (HWE *χ*^2^ < 10). SNPs also required a read depth of 30 × for inclusion. A total of 23 significant T2D-associated SNPs (*P* < 0.05), based on allelic *χ*^2^ tests for each SNP in the real NGS data using PLINK[10], were investigated by ‘masking’ them (i.e. removing them from the data) and imputing them using the remaining NGS SNPs as ‘scaffolds’. Details of the three regions are summarised in Table 1, and the genotypic data from all the NGS cases and control that were used in this study are provided in Additional file 2.

### Reference panel, imputation, and haplotype analysis

Imputation was carried out using IMPUTE2 [7] with the recommended default parameters. For all three comparisons, the cases and controls were pooled together to create the study panels, which were phased using SHAPEIT2 [28] using the default parameters. As recommended by IMPUTE2, the global list of haplotypes from the phased 1000 Genomes Project (1000GP) was used as the main imputation reference panel (b37, Oct 2014 release [29] , https://mathgen.stats.ox.ac.uk/impute/1000GP_Phase3.html). Genotypes were called from imputed data using GTOOL (http://www.well.ox.ac.uk/~cfreeman/software/gwas/gtool.html) with calling default threshold of probability 0.9. Standard single SNP association analyses (allelic *χ*^2^ tests) were carried out for the observed and inferred genotypes of all the T2D-associated SNPs using PLINK [10]. The performance of imputation was examined by comparing the observed and inferred genotypes for all three experiments that are shown in Fig. 1A–C. For the low-coverage experiment 1, we also used the case–control T2D NGS as the reference panel. Metrics such as info scores, missingness, and genotype discordance were collected after imputation. The info score is an information metric that ranges from 0 to 1, with values of ~ 1 indicative of no uncertainty in the imputed genotypes. The SNP data from the 92 cases and 93 controls were independently phased as one group using PHASE v2.1.1 [11] in order to conduct detailed comparisons of haplotypes between the study and reference panels. Haplotypes with posterior probabilities less than 90% were excluded from these comparisons (Fig. 1E).

### Supplementary Information

**Additional file 1: Table S1.** Detailed output from the imputation for the three different experiments as shown in Fig 1. **Table S2.** Comparison between the main reference panel (1000GP) and the NGS case-control data as the reference panel.

**Additional file 2. **In this file we provide the genotypes of all SNPs from the NGS T2D cases and controls and for all 3 loci that were used in this study.

**Additional file 3. **Review history.

## Data Availability

The dataset that generated this study is available in additional file 2. The IMPUTE2 software, the 1000GP phase 3 haplotypes that were used as the reference panel and the cM distances can all be found at [7]. The PHASE v2.1.1 software that was used to phase the data in additional file 2 could be found at [11].
