# Supplementary material for: The hazards of genotype imputation when mapping disease susceptibility variants
Source: Genome Biol. 2024 Jan 3;25:7. doi: 10.1186/s13059-023-03140-3 (PMC10763476; doi:10.1186/s13059-023-03140-3)
Supplement: Supplementary file 1 — Additional file 1: Table S1. Detailed output from the imputation for the three different experiments as shown in Fig 1. Table S2. Comparison between the main reference panel (1000GP) and the NGS case-control data as the reference panel. [file 13059_2023_3140_MOESM1_ESM.docx]

**Additional file 1: Supplementary tables S1 and S2**

**The hazards of genotype imputation when mapping disease susceptibility variants**

Winston Lau^1†^, Aminah Ali^1†^, Hannah Maude^2†^, Toby Andrew^2^_,_ Dallas M. Swallow^1^ and Nikolas Maniatis^1^*^*^*

^†^ Winston Lau, Aminah Ali and Hannah Maude contributed equally to this work.

__________________

^1^University College London, Department of Genetics, Evolution and Environment,

UCL Genetics Institute, London, UK

^2^Imperial College London, Section of Genetics and Genomics, Department of Metabolism, Digestion and Reproduction, London, UK

**^*^**Correspondence: [n.maniatis@ucl.ac.uk](mailto:n.maniatis@ucl.ac.uk)

**Additional file 1: Table S1: Detailed output from the imputation for the three different experiments**

All imputed genotypes are based on the 1000 Genomes Project (1000GP) reference panel. Red alleles are the dataset's original but changed after imputation to the alternative alleles. For example, for the rs13285616, the cases AG(3), GG(1) indicate that 3 AG became AA and 1 GG became AA. The red *NN* is the original missing genotype that changed to homozygous for the major allele.

**Additional file 1: Table S2: Comparison between reference panels**

The T2D-association statistic (*χ*^2^) for the observed and imputed SNPs. Those with ‘-‘ were imputed as monomorphic. Red alleles are the dataset's original but changed after imputation to the alternative alleles. For example, for the rs13285616, the cases AG(3), GG(1) indicate that 3 AG became AA and 1 GG became AA. The red *NN* is the original missing genotype that changed to homozygous for the major allele.
