# Supplementary material for: The hazards of genotype imputation when mapping disease susceptibility variants
Source: Genome Biol. 2024 Jan 3;25:7. doi: 10.1186/s13059-023-03140-3 (PMC10763476; doi:10.1186/s13059-023-03140-3)
Supplement: Supplementary file 3 — Additional file 3. Review history. [file 13059_2023_3140_MOESM3_ESM.docx]

**1^st^ round**

**Reviewer 1**

This is an insightful paper in which the authors delve deeper into the limitations of imputation in status quo methods. They build on previous work identifying 3 genes associating with type 2 diabetes (1 well-established and 2 novel). The authors demonstrate challenges in imputation that could explain why the 2 novel loci haven't been identified in previous studies. The authors provide a clear outline of why imputation is problematic and its limitation. They demonstrate clearly how different combinations of including GWAS significant signals as part of high/low density scaffold SNPs for imputation can produce disparate results.


Major comments
-The background does a great job highlighting the general challenges with imputation, but more specific details on the context of motivation for the scope of this particular investigation can be given for clarity, particularly between paragraphs of line 46 and line 58 on page 3
-the main takeaway is that larger reference panels are needed to do the LD calculation, and in an ancestry-specific way, so that the target and ref populations have similar LD substructure
-this is a known finding but the authors here demonstrate with more granularity why the care should be taken to assure this
-the main takeaway is that imputation errors result in changes in effect size, making the results of different GWAS and thus reducing replication rates and increasing false negatives
-a discussion of the other factors which may also be influencing this and have yet to be tested can be included in the discussion section


Specific comments:

-It's not clear in the background section (line 1 onwards page 4; line 45 page 4) that the 3 loci of interest come from the authors' previous study; please explicitly state that the motivation for this manuscript is that the novel loci were not identified in previous studies conducted by others. The cohort used to do the GWAS is referenced (5800 cases/9691 controls) but it was not clear that that analysis was done previously and not in this paper.
-Adding in a few lines in the methods stating the way in which the GWAS from the previous paper was done will help readers gain more context and won't require them to go back to the authors' other paper.
-A replication analysis showing that 3 loci were not significant in another GWAS (ex. UKBB) would significantly strengthen the paper and provide a concrete example of lack of replication
-Outline other reasons they could not replicate GWAS significant results i.e. allele frequency, population substructure, etc.
-An overarching schematic indicating the 3 analyses done and the input/output for each would be very helpful as a Figure 1 to orient the reader to the references made for each analysis throughout the paper
-An interpretation of Table 1 should be provided to give context of the findings
-How are the SNPs shown in table 2 selected? Were all statistically significant?
-it is unclear in table 3 if most of the SNPs were simply not a part of the 2020 meta-analysis, and if so, why
-figure 1 is comprehensive but a bit confusing as to what the main takeaway should be.
-similarly in figure 2, an interpretation of why the delta in genetic distances matter between analyses with/without imputation would be helpful - it is unclear to me what the main takeaway is

Minor:
-brief explanation of fine-mapping methods like "causal location estimation" (line 8 page 5)
-Is Burrows-Wheeler transform the standard for aligning human reference sequence? (line 25 page 5)
-define "LDU" earlier within background section
-define "scaffold SNPs" in study design section
-define "masking" in study design section
-define "mono/polymorphic" allele
-define "mono/polyphyletic"
-Monophyletic has the connotation of a common origin across organisms in the phylogeny tree - the context of line 56 on page 9 is not clear - why does this suggest a monophyletic origin and what is the implication of this? Line 16 page 10 also does not provide clarity on why this is a polyphyletic origin of T2D other than based on the LD map

**Reviewer 2**

Lau et al. investigate whether the widely used method of genotype imputation is risk-free. After analyzing a case study of NGS data, the authors conclude that a paradigm shift is needed for meta-analysis across populations and omics data.

Overall:
This paper is a follow-up to the previous paper published by the same authors entitled: "The hazards of genotype imputation in chromosomal regions under selection: A case study using the Lactase gene region".
I agree with the authors that it is extremely important to understand and evaluate the limitations of genotype imputation - as many other studies have done, and this study shares interesting observations. However, the case study presented is too limited and focused on the (partially) known limitation of genotype imputation to draw such strong conclusions. Furthermore, the multi-marker method favored by the authors in this and the previous paper (e.g., Lau et al., 2017) is not systematically compared and evaluated.

Specific points:
- Methods part / Study design does not include enough details to replicate the work.
- The authors describe that the GWAS backbone was designed by mimicking the density of different chips - but this is not enough, the chips are specifically optimized for genotype imputation and therefore exactly the same variants have to be used for this experiment (and please report how many were available for analysis). Also, the regions analyzed are extremely small - to compare with a proper imputation setting, these regions need to be expanded (200-500kb).
- The authors used only the somewhat (outdated) 1000G panels. The cited Mahajan et al., 2022 paper not only used 1000G but also HRC and population specific panels. Moreover, for the most recent study (Suzuki K et al.,medRxiv [Preprint]. 2023 Mar 31:2023.03.31.23287839. doi: 10.1101/2023.03.31.23287839. PMID: 37034649; PMCID: PMC10081410.) also the TOPMed panel was used by many studies. Therefore, I recommend to re-run the analysis with some of the more recent reference panels (HRC or TOPMed or both).
- To support the general conclusions, this study would need to include association results for quantitative traits and other OMICS data.
- To account for genotype uncertainty, genotype dosage should be used in the analysis (Zheng et al., 2011). Why were only hard calls used? How much would the results change?
- Phasing affects the quality of genotype imputation, it seems that the authors used the data set phased with PHASE as a gold standard. It would be important to compare the quality of phasing with the SHAPEIT results for the different chip scales.
- In contrast to many other studies (e.g., Hanks et al., 2022), the authors did not observe a correlation between MAF and imputation quality. This could simply be due to the very small number of variants evaluated.
- In my opinion, this paper is partly unbalanced, it underestimates the fact that genotype imputation is the basis for the identification of 1000s of signals (https://www.ebi.ac.uk/gwas/), that many problems are well known and actively investigated in large studies, and that it provides only a very limited comparison with the favored imputation-free multi-marker method.

**Authors’ response**

Reviewer 1

**REVR:** This is an insightful paper in which the authors delve deeper into the limitations of imputation in status quo methods……The authors provide a clear outline of why imputation is problematic and its limitation. They demonstrate clearly how different combinations of including GWAS significant signals as part of high/low density scaffold SNPs for imputation can produce disparate results.

***AU:*** *It is pleasing to know that Reviewer 1 found our paper insightful and that we have provided a clear outline of the hazards of imputation in gene mapping.*

**REVR:** The background does a great job highlighting the general challenges with imputation, but more specific details on the context of motivation for the scope of this particular investigation can be given for clarity, particularly between paragraphs of line 46 and line 58 on page 3.

***AU:*** *This has now been addressed. We thank the reviewer for the good suggestion. We have added a new paragraph about the 3 motivations of the study, which includes investigation of haplotype differences between the study panel and the reference panel. We hope that this addition (2nd paragraph, p4 lines 8-20) makes the scope much clearer and it should also help with the Reviewer’s comment below.*

**REVR:** the main takeaway is that larger reference panels are needed to do the LD calculation, and in an ancestry-specific way, so that the target and ref populations have similar LD substructure this is a known finding but the authors here demonstrate with more granularity why the care should be taken to assure this

***AU:*** *We thank the reviewer for commenting on the benefits of the granularity of our study. Differences in LD according to ancestry is one aspect of our message but the other is that   any haplotype differences that exist between the study panel and the reference panel will lead to inaccurate imputation. This is of a major concern because* ***haplotype-differences between cases and controls are in fact expected in regions of true disease association****. This is why we advise against imputation when mapping disease susceptibility variants. We have addressed the Reviewer’s comment by a) making the motivation of the study clearer (see also comment above); b) rewriting the conclusions section where it is made clear that because of our findings on haplotype frequency differences, imputation should be avoided (p16 lines 16-37.*

**REVR:** the main takeaway is that imputation errors result in changes in effect size, making the results of different GWAS and thus reducing replication rates and increasing false negatives

***AU:*** *Yes indeed but we also identify the reason as to why we are getting these errors (main takeaway message above).*

**REVR:** a discussion of the other factors which may also be influencing this and have yet to be tested can be included in the discussion section

***AU:*** *This has now been addressed by adding a new paragraph at the end of the discussion section (p16 lines46- next page line 9) about future avenues for research work, focusing on the need to examine* the accuracy when phasing unrelated individuals from different ancestries in the current reference panels.

**REVR:** It's not clear in the background section (line 1 onwards page 4; line 45 page 4) that the 3 loci of interest come from the authors' previous study; please explicitly state that the motivation for this manuscript is that the novel loci were not identified in previous studies conducted by others. The cohort used to do the GWAS is referenced (5800 cases/9691 controls) but it was not clear that that analysis was done previously and not in this paper.

***AU:*** *This has now been addressed by rewriting page 4 – we deleted sentences that could potentially confuse the reader and made it clearer in the new text that these 3 loci were indeed identified by us previously and not as part of this study (p4 lines 23-47).*

**REVR:** Adding in a few lines in the methods stating the way in which the GWAS from the previous paper was done will help readers gain more context and won't require them to go back to the authors' other paper.

***AU:*** *This has now been addressed. We have added some detail about why the method we have previously used has the power to estimate causal locations (new paragraph in p4 lines 26-33). We felt that the added text on association mapping without imputation using linkage disequilibrium genetic maps is better suited to the introduction and discussion sections rather than in methods. We thank the Reviewer for this suggestion because although the research work we present herein is entirely on imputation and not on association mapping methods, this addition helps to connect better the imputation findings with our final figure in the discussion section.*

**REVR:** A replication analysis showing that 3 loci were not significant in another GWAS (ex. UKBB) would significantly strengthen the paper and provide a concrete example of lack of replication….

***AU:*** *We feel that this is not necessary for 2 reasons. Firstly, we replicated the observations about inaccuracies from imputation in all three regions, including the most potent signal and well-studied T2D gene, TCF7L2. Secondly, Table 3 compares published findings from 2 large-scale meta-analyses that fail to show significant association for many of the same SNPs and essentially this is our replication. We appreciate that the initial manuscript did not convey this as clearly as we had hoped, hence we have now addressed this by making it much clearer in the discussion section (new paragraph p14 lines 20-39).*

**REVR:** Outline other reasons they could not replicate GWAS significant results i.e. allele frequency, population substructure, etc.

***AU:*** *We have now addressed this in the discussion section and added the reason other than imputation (3rd paragraph, p13 lines 40-50), namely differences in methodology using fine-scale LD structure between ethnic groups.*

**REVR:** An overarching schematic indicating the 3 analyses done and the input/output for each would be very helpful as a Figure 1 to orient the reader to the references made for each analysis throughout the paper

***AU:*** *We have added a schematic of the components of the study.  We thank the reviewer for this suggestion (new Figure 1, p7).*

**REVR:** An interpretation of Table 1 should be provided to give context of the findings.

***AU:*** *Now done (1st paragraph, p5 lines 4-14, 36-40 and also 51-55) and we have also improved the text within Table 1 (please note that changes within the table do not show as track changes).*

**REVR:** How are the SNPs shown in table 2 selected? Were all statistically significant?

***AU:*** *Yes indeed, the SNPs in Table 2 were all the significant ones identified in our previous study. These are the ones that were used to examine imputation, by masking them in this study. They are also shown as black diamonds on Figure 4. Table 2 gives the chi-square value of their association with T2D (Column 7). We have addressed this comment by making it clearer in the legend of Table 2 and by updating Table 1, which summarises the data used, so that it is easier to follow the significant SNPs in both Table 2 and text.*

**REVR:** it is unclear in table 3 if most of the SNPs were simply not a part of the 2020 meta-analysis, and if so, why

***AU:*** *These SNPs were included in the 2020 meta-analysis but were not observed to be significant. We made an appropriate change within Table 3 to clarify this point (please note that changes within the table do not show as track changes).*

**REVR:** -figure 1 is comprehensive but a bit confusing as to what the main takeaway should be.

***AU:*** *We have addressed this by adding a take home message from this figure (now Figure 2) to the discussion (starting 2nd paragraph, p12 lines 18-26).*

**REVR:** similarly in figure 2, an interpretation of why the delta in genetic distances matter between analyses with/without imputation would be helpful - it is unclear to me what the main takeaway is

***AU:*** *We have also addressed this by adding the take home message of this figure (now Figure 3) to the discussion (p14 lines 32-39 and p15 lines 46-55).*

*.*

**REVR:** Brief explanation of fine-mapping methods like "causal location estimation" (line 8 page 5).  **I**s Burrows-Wheeler transform the standard for aligning human reference sequence? (line 25 page 5)

***AU:*** *We have added some notes about our method that estimates causal locations in the introduction (last paragraph, p4 lines 23-35). Yes Burrows-Wheeler transform is standard in many alignment tools for speeding up the matching process and find how many reads match the reference sequence and at what positions. We have also added in the text ‘at the time of publication’ (p5 line 21).*

**REVR:** -define "LDU" earlier within background section

-define "scaffold SNPs" in study design section

-define "masking" in study design section

-define "mono/polymorphic" allele

-define "mono/polyphyletic"

-Monophyletic has the connotation of a common origin across organisms in the phylogeny tree - the context of line 56 on page 9 is not clear - why does this suggest a monophyletic origin and what is the implication of this? Line 16 page 10 also does not provide clarity on why this is a polyphyletic origin of T2D other than based on the LD map

***AU_18:*** *All the above have been defined. Also, a short section has been added (last paragraph, p11 lines 9-15) that defines polyphyletic haplotypes and why the specific ones on T2D are polyphyletic and what they mean in relation to the findings.*

Reviewer 2

**REVR:** This paper is a follow-up to the previous paper published by the same authors entitled: "The hazards of genotype imputation in chromosomal regions under selection: A case study using the Lactase gene region".

I agree with the authors that it is extremely important to understand and evaluate the limitations of genotype imputation - as many other studies have done, and this study shares interesting observations.

***AU:*** *We are pleased to know that Reviewer 2 found our observations interesting.*

**REVR:** However, the case study presented is too limited and focused on the (partially) known limitation of genotype imputation to draw such strong conclusions.

***AU:*** *Strong conclusions were indeed drawn from our novel findings. The reviewer’s comments made us reread the manuscript and we noted that the conclusions section may have been a little unclear. We have now made important edits in the introduction, discussion and the conclusions that clarify the points that the reviewer seems to have misunderstood. Even though we investigated the impact of SNP density in relation to disease-associated variants as opposed to genome-wide error rates, this was only one aspect of our work.* ***The main novel finding from our paper is that the reference panel will tend to drive imputation towards making allelic calls that are more frequent in the reference panel, leading to inaccurate imputation in the case-control study panel at disease loci with different haplotype frequencies between cases and controls.*** *The strong conclusion is that we advise against imputation since if haplotype structure differs between cases and controls as we show here, then the reference panels may never be suitable. Please see edits in the introduction (p3 lines 26-33, p4 8-20), discussion (p12 lines 18-24) and the conclusions (p16 lines 15-24).*

**REVR:** Furthermore, the multi-marker method favored by the authors in this and the previous paper (e.g., Lau et al., 2017) is not systematically compared and evaluated.

- Methods part / Study design does not include enough details to replicate the work.

***AU: We are not using our multimarker method to examine imputation at all.*** *We analyse loci that we previously identified* ***without imputation*** *using our multimarker method but this manuscript has nothing to do with this method or any other mapping method.  Instead, it uses a widely implemented imputation software IMPUTE2 to compare standard single-SNP summary statistics before and after imputation and to compare independently phased haplotypes between the study panel and the reference panel. We have now addressed the reviewer’s comment by making it clearer in the text that these loci were found previously and not as part of this study (p4 lines 23-47). We added a schematic diagram of our study design that shows step-by-step what we did so that it could be very easily replicated (Figure 1, p7). We made it clearer in the methods that we have used standard single SNP-by-disease associate tests for the both the real genotypes (p5, lines 38-40) and imputed ones (p6, lines 53-55) so that others could replicate using the default parameters of IMPUTE2.*

**REVR:** The authors describe that the GWAS backbone was designed by mimicking the density of different chips - but this is not enough, the chips are specifically optimized for genotype imputation and therefore exactly the same variants have to be used for this experiment (and please report how many were available for analysis). Also, the regions analyzed are extremely small - to compare with a proper imputation setting, these regions need to be expanded (200-500kb).

***AU:*** *Yes, we did indeed mimic the density of an early Affymterix GWAS chip for our first comparison (Table 2). But the second and third comparisons in Table 2 however, did not mimic any GWAS and instead used all the NGS data – a far denser marker panel than the 2nd generation panels used by other studies (see Table 1 for this comparison) and hence more favourable for imputation. We were puzzled at first with the comment that SNP arrays are optimised for imputation. We examined the Illumina ~2.5M SNP array (link below) but did not find anything about optimisation for imputation.*

<https://www.illumina.com/products/by-type/microarray-kits/infinium-omni25-8.html>

*As far as we know, there have not been systematic strategies for tag-SNP selection methods based specifically on imputation quality (imputation methods being developed more recently). We assume therefore, that the Reviewer meant that the current SNP arrays offer optimised tag-SNP content for population-specific LD blocks from reference panels. But for our investigation we used very high resolution NGS data anyway, which surpasses the SNP density of the Omni 2.5M array. We have made the text clearer about this when interpreting the data in Table 1. Further, using exactly the same variants as the SNP array is not feasible not only for our study, but for all other studies, because different GWASs have used different SNP arrays and each GWAS is subject to its own quality control for Hardy-Weinberg, missingness etc.*

*Also, the main motivation of our study was to reconstruct haplotypes for our T2D cases and controls and compare these with the haplotypes from the reference panel. Haplotypes are unquestionably more accurate in shorter regions. These three loci are flanked by recombination hotspots so expanding our regions to 200-500 kb would have made it impossible to draw accurate conclusions about the differences in haplotype diversity between study and reference panels and the impact they had on imputation accuracy.  We have addressed the Reviewer’s comment by explaining our motivation behind short haplotypes as opposed to long-range haplotypes (p4 lines 15-20, p5 lines 7-14) and also making changes in the discussion section, where we also discuss the accuracy of phasing (cases/controls  and reference panels) that is based in unrelated individuals (p16 lines 1-8).*

**REVR:** The authors used only the somewhat (outdated) 1000G panels. The cited Mahajan et al., 2022 paper not only used 1000G but also HRC and population specific panels. Moreover, for the most recent study (Suzuki K et al.,medRxiv [Preprint]. 2023 Mar 31:2023.03.31.23287839. doi: 10.1101/2023.03.31.23287839. PMID: 37034649; PMCID: PMC10081410.) also the TOPMed panel was used by many studies. Therefore, I recommend to re-run the analysis with some of the more recent reference panels (HRC or TOPMed or both).

***AU:*** *Yes this is true, but GWAS results are not becoming obsolete (e.g. the large T2D 2020 study^5^ used only 1000GP and the Mahajan study^6^ did not use TOPMed) and studies are meta-analysing single imputed SNP summary statistics that are derived from different reference panels, including HapMap. But the main and important issue is that imputation is based upon a set of inferred haplotypes and that haplotype differences are (by definition) expected between* ***ascertained*** *cases (disease GWAS) and reference panels at disease loci with true positive associations.* ***Thus, our conclusion is about the methodological foundation of imputation itself and not about the reference panel per se, because none of these panels will ever include patients ascertained for the same disease and ancestry as the disease GWAS in question.*** *Also, the haplotypes in both 1000GP or TOPmed are inferred from unrelated individuals, which means that both phasing and also imputation depend on the intensity of historical recombination, which varies across populations. We have explicitly addressed this in the MS by making changes in our discussion section (in relation to TOPMed, p12 lines 40-55) and also in the section about future work (p16 lines 1-8).*

**REVR:** To support the general conclusions, this study would need to include association results for quantitative traits and other OMICS data.

***AU:*** *We appreciate the comment but if the imputed genotypes have inaccuracies, then imputation will impact any type of association study and will be true for both quantitative and qualitative traits. The error lies with the inferred genotype prior to any test of association. Our study is specific to disease gene mapping, but we have addressed this comment in relation to the challenges of imputation when co-locating disease loci with eQTL mapping using OMICS data (p15 58-60).*

**REVR:** To account for genotype uncertainty, genotype dosage should be used in the analysis (Zheng et al., 2011). Why were only hard calls used? How much would the results change?

***AU:*** *The study by Zheng et al (2011) is on how to take into account uncertainty in imputed genotypes when testing association with disease.* ***But weighting for uncertainty does not address what we study here, which is the cause of imputation errors.*** *Here we find that imputation certainty is heavily impacted by true positive differences of haplotype frequencies between cases and reference panel. It is therefore reasonable to conclude that the results from such methods using weighted tests of association will be impacted too. Also, we chose to use standard statistical tests for disease-by-SNP association so that we can compare our findings with large-scale published results on T2D that did not used the method by Zheng et al (2011). In addition, we present imputed SNPs in Table 2 with high certainty scores but highly discordant with the true genotypes too. For example, 0.95 score for ACTL7B (low-density, column 9) and 0.77 score for TCF7L2 (high-density, column 11). Also, we provide supplementary tables that present discrepancy count data to identify how and why the discrepancies in genotype calls actually arise.  We have addressed the Reviewer’s comment by making the scope of the study (p4 lines 8-20) and methods sections clearer. Regarding the Reviewer’s comment about hard calls, the answer is because 0.9 is the default threshold given by the authors of IMPUTE2 software:* <https://mathgen.stats.ox.ac.uk/impute/impute_v2.html#-known_haps_g>

*and we followed their instructions carefully. We have now made it clearer in the text that this is the default. However, we also tried 0.8 but we obtained similar results.*

**REVR:** Phasing affects the quality of genotype imputation, it seems that the authors used the data set phased with PHASE as a gold standard. It would be important to compare the quality of phasing with the SHAPEIT results for the different chip scales.

***AU:*** *We did use SHAPEIT with IMPUTE2, but we used PHASE for reconstructing haplotypes of our study panel. This is because PHASE has been shown to be the most accurate in shorter regions^27^. We have addressed this by explaining this in the methods section and also citing references that demonstrate this (p5 lines 8-11, p6 lines 43-45, p7 lines 1-9).*

**REVR:** In contrast to many other studies (e.g., Hanks et al., 2022), the authors did not observe a correlation between MAF and imputation quality. This could simply be due to the very small number of variants evaluated.

***AU:*** *We thank the reviewer for this comment and for pointing out this paper. Hanks et al used much smaller MAFs. For our three gene regions, the SNPs ranged from 0.1-0.5 (Table 2, column 4).  Also, note that Hanks concluded that “***Imputation quality also varied across genomic regions, producing regions where even common (MAF > 5%) variants were consistently not well imputed across ancestries***”. Their finding supports our argument that we need to examine imputation in disease-associated regions rather than just looking genome-wide imputation error rates.  It is important to emphasise that in disease GWAS are highly ascertained. We have expanded the discussion and included this discussion point by citing Hanks et al.^30^ (p12 lines 43-55).*

**REVR:** In my opinion, this paper is partly unbalanced, it underestimates the fact that genotype imputation is the basis for the identification of 1000s of signals (https://eur01.safelinks.protection.outlook.com/?url=https%3A%2F%2Fwww.ebi.ac.uk%2Fgwas%2F&data=05%7C01%7Cn.maniatis%40ucl.ac.uk%7C93731fa7c09145991a7408db6c2b58ae%7C1faf88fea9984c5b93c9210a11d9a5c2%7C0%7C0%7C638222705659320849%7CUnknown%7CTWFpbGZsb3d8eyJWIjoiMC4wLjAwMDAiLCJQIjoiV2luMzIiLCJBTiI6Ik1haWwiLCJXVCI6Mn0%3D%7C3000%7C%7C%7C&sdata=U3c86nD4%2F2IxsQakeP4ATuEvNcp%2F82yhzSIs8d8DJbE%3D&reserved=0), that many problems are well known and actively investigated in large studies, and that it provides only a very limited comparison with the favored imputation-free multi-marker method.

***AU:*** *We appreciate the comment and this is why we have investigated the GWAS published lead SNPs (Figure 3, Table 3).  We found that the published signals are primarily in regions of strong LD, which we demonstrate in Figure 3. These regions are expected to contain some directly genotyped markers (non-imputed) because as the Reviewer 2 pointed out above, the array platforms are tagging LD blocks. But this finding is also telling us that missing genotypes are also more likely to be correctly imputed in regions of very strong LD. This is because haplotypes in the reference panel can be reconstructed with less error in such regions. Nevertheless, the Odds Ratios even for these lead SNPs are very low compared to directly genotyped ones in our study and elsewhere. Inaccuracies due to imputation are still an issue for these GWAS catalogues because of low reproducibility of these lead SNPs across ancestries with similar disease prevalence despite using global-population reference panels. We have addressed this issue in the discussion section where we made the interpretation of our findings clearer and more detailed (revised p14 lines 21-60).*

**2^nd^ round**

**Reviewer 2**

The authors have greatly improved the clarity of the manuscript by rewriting several parts and addressing several of my comments.

However, one of the main findings of this study
“Here we show that haplotype differences between the study panel and the reference panel drove imputation towards making allelic calls that are more frequent in the reference panel, than in the case-control study”

has been somewhat described and discussed, for example, in [DOI: 10.1146/annurev-genom-083117-021602]

“Additionally, disease-specific studies often benefit from a hybrid approach that combines a custom reference panel with an existing public reference panel. This approach is particularly beneficial for disease studies because it significantly increases the accuracy of imputing a causal variant (since the hybrid panel is enriched with more copies of rare alleles). One example highlighting this feature is a recent study on prostate cancer that was eventually able to impute the rare HOXB13 G84E variant only after using a hybrid imputation approach that combined the 1000G data with an enriched set of cases carrying the mutation“

and another example is this recent study on cystic fibrosis [DOI: 10.1016/j.xhgg.2022.100090]

“We further demonstrate the value of a CF-specific reference panel, which can outperform TOPMed for some variants owing to better match with target (also CF) samples in terms of allele and haplotype frequencies. Although at the 1-Mb region level, a CF-specific reference panel never outperformed the TOPMed reference panel, in some regions, it offers substantially more complementary information to TOPMed. These regions include the CFTR region harboring the gene causing this Mendelian disease, and several other genome regions including HLA. “

Nonetheless, I see value in a dedicated paper describing this issue; however, given the previous work, it would be important to repeat the analysis using, for example, the TOPMed reference panel, which includes not only healthy individuals. Even accepting the authors' response that genotype error caused by haplotype mismatch will also affect quantitative trait analysis, it would greatly increase the value of this paper to perform an analysis for a quantitative trait using also genotype dosage and show its effect (see also my original comments).

**Authors’ response**

R2: The authors have greatly improved the clarity of the manuscript by rewriting several parts

and addressing several of my comments.

AU: We thank the Reviewer for acknowledging the great improvement of our manuscript but

we consider that we addressed all previous comments and not just “several” of these.

R2: However, one of the main findings of this study

“Here we show that haplotype differences between the study panel and the reference panel

drove imputation towards making allelic calls that are more frequent in the reference panel,

than in the case-control study” has been somewhat described and discussed, for example, in

[DOI: 10.1146/annurev-genom-083117-021602]

“Additionally, disease-specific studies often benefit from a hybrid approach that combines a

custom reference panel with an existing public reference panel. This approach is particularly

beneficial for disease studies because it significantly increases the accuracy of imputing a

causal variant (since the hybrid panel is enriched with more copies of rare alleles). One

example highlighting this feature is a recent study on prostate cancer that was eventually able

to impute the rare HOXB13 G84E variant only after using a hybrid imputation approach that

combined the 1000G data with an enriched set of cases carrying the mutation“

AU: We disagree.

There is nothing in common between the quotation from our study and the quotation from Das

et al 2018 (a review article and not original research). The Das et al review, which we cite in

our introduction, differs from our study in scope, methods and findings.

Das et al suggest the use ‘hybrid panels’ for more accurate imputation by enriching a standard

reference panel (e.g. the 1000GP) for disease alleles. Such methods to improve the accuracy

of imputation is indeed not a novel topic and different strategies/data have been previously

proposed by others. But our study does not research such questions. Instead, it finds how and

why incorrect imputation inferences arise. Das et al did not investigate this and nor, as far as

we know, has any other paper on imputation.

Our investigation identified important differences in the haplotype structure between the

independently phased cases in the study and the reference panel. As such, the use of a hybrid

panel will not solve this problem because the frequency of haplotypes in the final hybrid panel

will still be different to the study haplotypes leading to inaccurate imputation of the patients’

missing genotypes. This is of major concern because haplotype-differences between cases

and controls are unquestionably expected in regions of true disease association, hence why

we advise against imputation at disease loci.

Importantly, our conclusion (first quotation) was strongly supported by the additional analyses “We further demonstrate the value of a CF-specific reference panel, which can outperform

TOPMed for some variants owing to better match with target (also CF) samples in terms of

allele and haplotype frequencies. Although at the 1-Mb region level, a CF-specific reference

panel never outperformed the TOPMed reference panel, in some regions, it offers substantially

more complementary information to TOPMed. These regions include the CFTR region

harboring the gene causing this Mendelian disease, and several other genome regions

including HLA. “

R2: and another example is this recent study on cystic fibrosis [DOI:

10.1016/j.xhgg.2022.100090]

“We further demonstrate the value of a CF-specific reference panel, which can outperform

TOPMed for some variants owing to better match with target (also CF) samples in terms of

allele and haplotype frequencies. Although at the 1-Mb region level, a CF-specific reference

panel never outperformed the TOPMed reference panel, in some regions, it offers substantially

more complementary information to TOPMed. These regions include the CFTR region

harboring the gene causing this Mendelian disease, and several other genome regions

including HLA. “

AU: We disagree.

Again, the Cystic Fibrosis (CF) paper by Sun et al cited does not reconstruct and compare

haplotypes as we have done and it focuses on improving the accuracy of imputation by

replacing TOPMed reference with their own CF-specific data. The authors have only

speculated that differences in haplotype frequencies explain their results by stating in the

abstract: “likely owing to allele and haplotype differences between patients with CF and

general populations”. But it is precisely the difference in disease haplotype frequencies and

how these give rise to imputation error that we explicitly analyse and quantify in our study

for the first time. As we pointed out above, unlike Sun et al, using our own T2D case-control

data as a highly informative reference panel with 50% T2D patients did not improve imputation

(Supplementary Table S2). But there is a fundamental difference between the Sun et al study

and our investigation. We are dealing with T2D, which is a polygenic disease (small effect

sizes) and not with CF, which is a Mendelian disease (large effect sizes). Nevertheless, we

believe that the strategy of obtaining disease-specific reference panels is not a viable one, not

only because of our findings, but also because it would defeat the purpose of imputation if we

require disease- and population-specific reference panels for every polygenic disease and

trans-ancestry GWAS. We have addressed the Reviewer’s comment by adding these

discussion points (pages 13, 14).

R2: Nonetheless, I see value in a dedicated paper describing this issue; however, given the

previous work, it would be important to repeat the analysis using, for example, the TOPMed

reference panel, which includes not only healthy individuals. Even accepting the authors'

response that genotype error caused by haplotype mismatch will also affect quantitative trait

analysis, it would greatly increase the value of this paper to perform an analysis for a

quantitative trait using also genotype dosage and show its effect (see also my original

comments).

AU: We disagree.

We have already responded in detail to the Reviewer’s original comment about quantitative

traits (please see our previous responses). The Reviewer’s suggestion to use TOPmed

contradicts the Reviewer’s decision to cite the above Sun et al study that concluded: “We

demonstrate that despite having approximately 3% the sample size of TOPMed, our CF

reference panel can still outperform TOPMed when imputing some CF disease-causing

variants”. As we have pointed out above, we have already used our own T2D reference panel

and not just the 1000GP alone. The Reviewer seems to have missed this analysis

(Supplementary Table S2). We have now addressed this by adding this important detail in the

methods section (page 6) and not just the discussion (pages 13, 14). We have also made

scope of our study clearer in order to avoid confusion about overlap with other published

studies on how to improve imputation errors or account for these (pages 3, 4).
